# The Complex Genetic Legacy of Hybridization and Introgression between the Rare *Ocotea loxensis* van der Werff and the Widespread *O. infrafoveolata* van der Werff (Lauraceae)

**DOI:** 10.3390/plants13141956

**Published:** 2024-07-17

**Authors:** David Draper, Lorena Riofrío, Carlos Naranjo, Isabel Marques

**Affiliations:** 1 Center for Ecology, Evolution, and Environmental Changes & CHANGE—Global Change and Sustainability Institute, Universidade de Lisboa, 1749-016 Lisboa, Portugal; 2 Facultad de Ciencias Exactas y Naturales, Universidad Tecnica Particular de Loja (UTPL), Loja 1101608, Ecuador; mlriofrio@utpl.edu.ec (L.R.); cjnaranjo@utpl.edu.ec (C.N.); 3 Forest Research Centre, Associate Laboratory TERRA, School of Agriculture, University of Lisbon, 1349-017 Lisbon, Portugal

**Keywords:** hybridization, Lauraceae, neotropical forests, plant diversity, tropical trees, speciation

## Abstract

Hybridization and introgression are complex evolutionary mechanisms that can increase species diversity and lead to speciation, but may also lead to species extinction. In this study, we tested the presence and genetic consequences of hybridization between the rare and Ecuadorian endemic *O. loxensis* van der Werff and the widespread *O. infrafoveolata* van der Werff (Lauraceae). Phenotypically, some trees are difficult to identify, and we expect that some might in fact be cryptic hybrids. Thus, we developed nuclear microsatellites to assess the existence of hybrids, as well as the patterns of genetic diversity and population structure in allopatric and sympatric populations. The results revealed high levels of genetic diversity, even in the rare *O. loxensis*, being usually significantly higher in sympatric than in allopatric populations. The Bayesian assignment of individuals into different genetic classes revealed a complex scenario with different hybrid generations occurring in all sympatric populations, but also in allopatric ones. The absence of some backcrossed hybrids suggests the existence of asymmetric gene flow, and that some hybrids might be more fitted than others might. The existence of current and past interspecific gene flow also explains the blurring of species boundaries in these species and could be linked to the high rates of species found in *Ocotea*.

## 1. Introduction

Breeding between species, i.e., interspecific hybridization, has often been coined as a negative process, leading in the best-case scenario to the blur of discriminant morphological features, and in the worst case to species extinction [1,2,3,4]. Accumulation of deleterious alleles, outbreeding depression, gamete waste and genetic swamping are among the detrimental consequences of hybridization, especially when involving rare species [5,6]. Yet, with the advent of genomic techniques, it became clear that many organisms show evidence of genetic admixture, and that hybridization could also have positive outcomes with the potential to foster novel adaptive traits [7,8,9,10]. In fact, despite the negative impacts of hybridization, a review based on all IUCN Red Data assessments found that it was only mentioned as a threat in 11 out of the 120,369 species assessed [11]. Among many biases, the idea of hybridization as a threat was found to be quite subjective in most of the assessments made, since there were no specific guidelines for quantifying the degree of threat deriving from hybridization [11]. In the opposite direction, possible benefits for the conservation of species deriving from hybridization are usually not considered [12,13]. Thus, determining the consequences of hybridization—either positive or negative—is crucial to understand the impacts that hybridization might have, and to tackle the causes of biodiversity loss.

Independently of the outcomes, hybridization appears to be a widespread process in plants, since about 25% of the known species hybridize naturally [14]. The number of reported hybrids is considerably high in well-studied temperate regions [15], with many specific studies detecting the complex consequences of hybridization, introgression, and hybrid speciation, as well as the challenges beyond hybridization [13,16,17,18,19]. Other studies evaluate the power of the analytic methods to detect the significance of the process itself [20]. In contrast, hybridization has received less attention in tropical environments, which has often led to the idea that the process is rare [21,22,23].

The Neotropical region is one of the most species-rich areas on Earth, encompassing many different biomes such as seasonally dry forests, arid zones, high-elevation grasslands, mountain systems, and extensive rainforests [24]. Many factors, such as the wide environmental and climatic heterogeneity, a complex geological history, together with ecological interactions and human impacts, have shaped the exceptional biodiversity found in the Neotropical region [25,26]. What remains less clear, however, is the role that hybridization might have played in the known Neotropical diversity. In fact, a recent review highlighted the need for more studies focused on hybridization in the Neotropical region [27] to understand the remarkable diversity found, where most species even lack a scientific name [28]. This review found only 60 plant studies dealing with hybridization in the Neotropics and concluded that outcomes due to hybridization had neutral effects in 50% of all cases, 45% showed positive effects, and only 5% showed negative effects [27]. Results of hybridization in Neotropical plants included rapid diversification events in several Andean groups, e.g., *Espeletiinae* [29], *Diplostephium* [30], *Polylepis* [31], *Lachemilla* [32], or *Vriesea* [33], reinforcement of reproductive barriers in *Costus* [34] and *Pitcairnia* [35], and the modification of morphological features, originating new lineages in *Epidendrum* [36,37]. However, outcomes might also include negative effects such as genetic swamping or the loss of barriers, which have the potential to reshape species interactions and lead to ecological shifts and new biotic relationships [5,27,37,38]. Thus, understanding the contribution of hybridization to the biodiversity of the Neotropics, one of the most species-rich areas on Earth, is a fundamental issue to understanding patterns of species distribution, richness, and endemism, as well as the risk of extinction.

**Figure 1:**
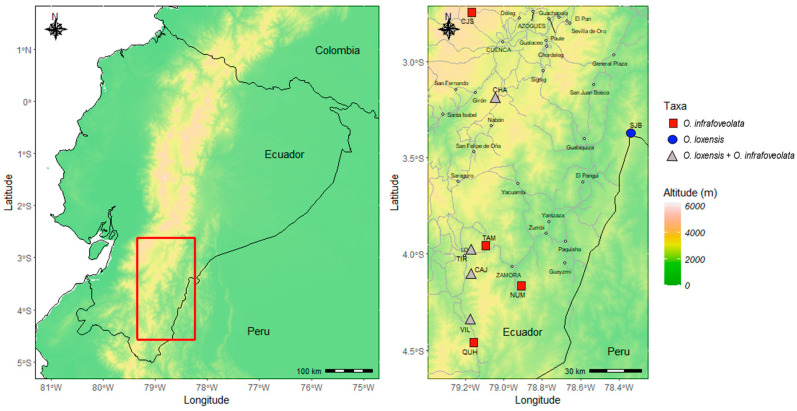
Topographic map of the study area in Ecuador with elevation displayed. Left: location of the studied area. Right: detail of the populations sampled, indicating allopatric populations of *O. loxensis* (blue circles), allopatric populations of *O. infrafoveolata* (red rectangles), and sympatric populations where the two species co-occur (gray triangles). Main roads are indicated by a dashed line. Main cities are also indicated.

In this study, we investigated the presence and degree of interspecific gene flow in two tropical trees from the Lauraceae family that co-occur in some populations in the South of Ecuador: *Ocotea loxensis* van der Werff and *O. infrafoveolata* van der Werff (Figure 1). *Ocotea loxensis* is a rare species, endemic to the South of Ecuador where it has a very restricted and scattered distribution, while *O. infrafoveolata* is a widely distributed species occurring from Colombia to North Peru. *Ocotea loxensis* can be misidentified as *O. infrafoveolata.* It can have smaller leaves (3–6 vs. 6–15 cm), flowers (6–7 vs. 7–10 mm diameter), tepals (2.5. vs. 3.5 mm), fruits (0.5 vs. 3 cm), and cupules (7–8 vs. 19 mm diameter) than *O. infrafoveolata*, although phenotypically some trees might indeed be hard to identify, especially in populations where more than one species occurs. Some trees also lack the decurrent revolute base of the leaves that clearly characterize *O. infrafoveolata* [39]. Given the close morphological traits and the fact that these species occur in some populations in the South of Ecuador, we tested the hypothesis that hybridization might occur between them and that cryptic hybrids might exist in sympatric populations, contributing to the blurring of morphological traits or genetic erosion. To achieve this aim, we genetically characterized populations of *O. loxensis* and *O. infrafoveolata* developing nuclear microsatellites to (1) understand the genetic diversity and structure of populations; (2) determine the presence of genetic admixture between species and if so, (3) the degree of asymmetrical hybridization events and the possible genetic outcomes. Overall, these findings provide new insights into the mechanisms and evolution of *Ocotea* species and contribute to explaining the high biodiversity of species found in Ecuador.

## 2. Results

### 2.1. Genetic Diversity of Loci

For each locus, the mean number of alleles varied between 1.385 in Oinf4 and 5.077 in Olox9, while the number of effective alleles varied between 1.201 in Olox3 and 3.732 in Olox9 (Table 1). Heterozygosity values also varied considerably between loci. For example, observed heterozygosity varied between 0.160 in Oinf20 and 0.884 in Olox1, while expected heterozygosity varied between 0.218 in Orot21 and Oinf5. The Polymorphism Information Content was very high in all loci, varying between 0.782 and 0.905. No null alleles or significant departures from HWE were detected. Pairwise comparisons between loci showed no significant disequilibrium (*p* > 0.05), revealing that all loci were assorted independently at the different loci.

### 2.2. Genetic Diversity and Differentiation in O. loxensis and O. infrafoveolata

In *O. loxensis*, the mean number of alleles (Na) varied between 3.733 in LOX-SJB and 4.260 in LOX-CHA, while in *O. infrafoveolata*, Na varied between 2.067 in INF-CHA and 4.488 in INF-TIR (Table 2). However, in both species, Na was significantly higher in sympatric than in allopatric populations, probably as a consequence of interspecific gene flow. The same pattern was recorded for the number of effective alleles (Ne) and Shannon’s information index (I). In *O. loxensis*, observed heterozygosity (Ho) values varied from 0.487 to 0.741 while the expected heterozygosity (He) varied between 0.518 and 0.692, respectively, in LOX-SJB and LOX-VIL (Table 2). Heterozygosity values were higher in *O. infrafoveolata* than in *O. loxensis*. However, they were always higher in sympatric than in allopatric populations. In *O. loxensis*, Fis values varied between −0.002 (LOX-CHA) and 0.0014 (LOX-SJB), while in *O. infrafoveolata* they varied between −0.848 (INF-CHA) and −0.030 (INF-TIR). The likely exchange of gene flow between the two species also affected the percentage of polymorphic loci, which was very high in the two species, but always higher in sympatric than in allopatric populations (Table 2).

Pairwise genetic differentiation coefficient values (Fst) among all populations ranged from 0.008 to 0.550 (*p* < 0.001; Table 3). The highest Fst values were observed between *O. loxensis* and *O. infrafoveolata* populations, especially when considering the two “pure” reference populations. These populations also showed a high degree of differentiation from the remaining *O. infrafoveolata* populations. Within *O. loxensis* populations, LOX-SJB also showed the highest level of differentiation when compared to the other populations.

The analysis of molecular variance (AMOVA) between all samples found that the highest level of variation was found within rather than among populations (Table 4). The fixation index was 0.101. When the AMOVA was performed considering only *O. loxensis* samples, 94% of the total variation was found within populations and the remainder was found among populations. The fixation index was 0.022. Yet, when only *O. infrafoveolata* samples were considered, the level of variation within populations dropped to 41% while the remaining 59% of variation was explained among populations. The fixation index was 0.063, much higher than that reported for *O. loxensis*, suggesting a higher genetic differentiation in *O. infrafoveolata*.

### 2.3. Genetic Structure of O. loxensis and O. infrafoveolata Populations

**Figure 2:**
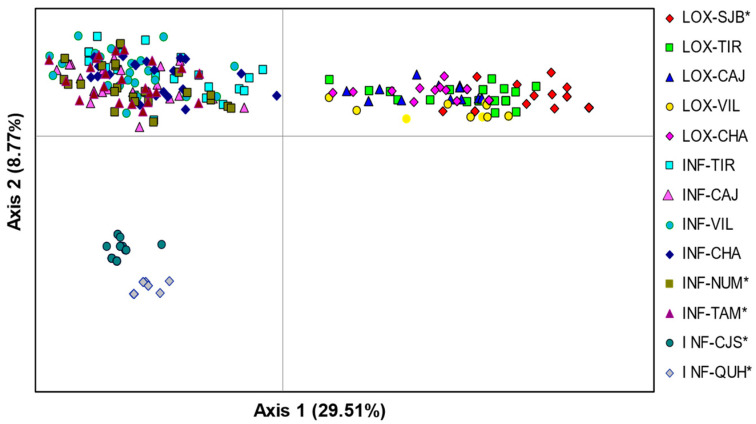
Genetic relationships between *O. loxensis* and *O. infrafoveolata* samples based on a principal coordinate analysis (PCoA). Population labels refer to Figure 1. * indicates allopatric populations.

The PCoA revealed three differentiated groups where axis 1 separated all individuals of *O. loxensis* from *O. infrafoveolata*, and axis 2 separated two allopatric populations of *O. infrafoveolata* (INF-CJS, INF-QUH) from all remaining populations of this species (Figure 2). There was also a slight spatial differentiation of the allopatric *O. loxensis* population (LOX-SJB) from the remaining populations of this species. The first two axes of the PCoA accounted for a high proportion of the total variance (53.26%), with 29.51% explained by the first axis and 8.70% by the second (Figure 2).

**Figure 3:**
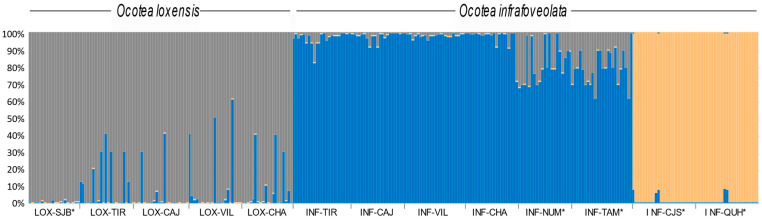
Genetic structure of *O. loxensis* and *O. infrafoveolata* samples based on the best assignment results retrieved by STRUCTURE (K  =  3). Each sample is represented by a thin vertical line divided into K-colored segments that represent the individual’s estimated membership fractions in K clusters. Population labels refer to Figure 1. * indicates allopatric populations.

The most likely number of clusters retrieved by STRUCTURE for the entire data set was *K* = 3, allocating all individuals of *O. loxensis* to a single cluster and most *O. infrafoveolata* individuals to a second cluster, while the individuals from the allopatric populations of INF-CJS and INF-QUH were characterized by a third cluster (Figure 3). Admixture was frequent, especially in the sympatric populations of *O. loxensis*. By contrast, evidence of genetic admixture in *O. infrafoveolata* was very low except in INF-NUM and INF-TAM populations (Figure 3).

### 2.4. Genetic Composition of Populations

**Figure 4:**
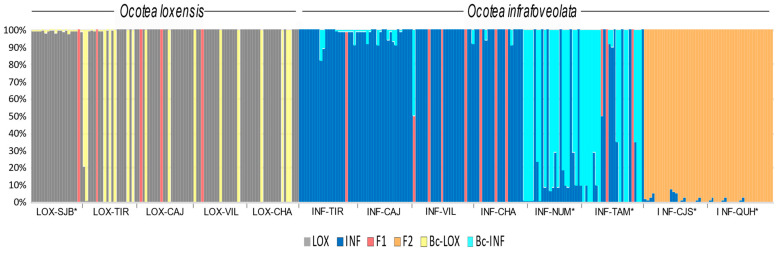
Genetic composition of *O. loxensis* and *O. infrafoveolata* samples based on NEWHYBRIDS. The proportion of color in each bar indicates the assignment probability according to the different genetic classes (pure parental species, F1, F2, and late-generation hybrids, and the respective backcrosses). Population labels refer to Figure 1. * indicates allopatric populations.

NEWHYBRIDS suggested that 95% of the sampled *O. loxensis* individuals in the allopatric population of LOX-SJB were pure while sympatric populations were composed of pure individuals (76.2%), backcrossed plants towards *O. loxensis* (19.0%) and F1 hybrids (4.8%; Figure 4).

In *O. infrafoveolata*, sympatric populations were mainly composed of pure individuals and F1 hybrids, although in a minor number (respectively, 90.9% and 9.1%; Figure 4). However, different results were found in allopatric populations. For instance, INF-NUM and INF-TAM populations that were characterized by the second K cluster in STRUCTURE were suggested to be mainly composed of backcrossed individuals towards *O. infrafoveolata* (51.0%), pure individuals (44.7%), and F1 hybrid generations (4.3%; Figure 4). The populations of INF-CJS and INF-QUH populations that were characterized in STRUCTURE by the third K cluster were suggested to be composed of F2 and late-generation hybrids (Figure 4).

Gene flow (Nm) was estimated to be greater from *O. loxensis* to *O. infrafoveolata* (3.230) than in the opposite direction (1.025).

## 3. Discussion

Comparisons of genetic diversity between rare and widespread related species can provide valuable information concerning the causes of rarity, and play a critical role in guiding conservation efforts [41]. Usually, genetic diversity is expected to be lower in a species that is not widely distributed compared with its widespread congener due to lower population numbers [42]. This limitation in potential mates could lead to a decrease in breeding, or breeding between close relatives, which altogether would decrease the level of genetic diversity [43]. The occurrence of population bottlenecks would also cause a significant reduction in the effective population size, the loss of rare alleles, and heterozygosity in the population [44,45]. Yet, in this study, we found no evidence of lower genetic levels in the rare *O. loxensis* in comparison with the widespread *O. infrafoveolata*. For instance, the number of effective alleles was similar between the two species (Ne = 2.75 and 2.72, respectively in *O. loxensis* and *O. infrafoveolata*), as well as the mean heterozygosity values (Ho = 0.67 and 0.69; He = 0.62 and 0.55, respectively in *O. loxensis* and *O. infrafoveolata*; Table 2). Mean inbreeding depression met neutral expectations in *O. loxensis* (Fis = −0.01), being positive only in the sympatric population, while negative values were found in all *O. infrafoveolata* populations (mean Fis = −0.339), suggesting an excess of heterozygotes. The fixation index of *O. infrafoveolata* was also much higher than the one reported for *O. loxensis* (0.063 vs. 0.022, respectively) revealing a higher degree of population differentiation, probably due to genetic structure (Figure 4). In fact, although the partitioning of genetic variation was high within populations in both species, levels were lower in *O. infrafoveolata* (41% vs. 94% in *O. loxensis*; Table 4). The percentage of polymorphic loci was also very high in both species. Thus, the restricted distribution of *O. loxensis*, probably due to a historical range reduction in the past, showed no effect on genetic diversity when compared with *O. infrafoveolata* (at least when considering all populations). These high levels of genetic diversity could be explained by high outcrossing rates between populations, which would explain the absence of a spatial structure associated with genetic data (Figure 2 and Figure 3).

The diversity values reported here were slightly similar to the ones found in *O. rotundata*, a highly fragmented species known only from five fragmented patches, in the South Ecuadorian provinces of Loja and Zamora-Chinchipe [40]. In that study, the mean heterozygosity was reported as Ho = 0.652 and He = 0.76, despite the high number of alleles found, i.e., 9.84 alleles [40]. Nevertheless, the observed heterozygosity values reported in this study were higher than the ones found in *O. odorifera* (Vell.) Rohwer (Ho = 0.63), *O. catharinensis* Mez (Ho = 0.57), and especially in *O. porosa* (Nees & Mart.) Barroso (Ho = 0.52), three species severely threatened by overexploitation [46]. The values reported here were also higher than the heterozygosity values found in other species within the family Lauraceae, such as *Litsea auriculata* S.S. Chien & W.C. Cheng (Ho = 0.33 to 0.50; [47] or *Cinnamomum balansae* Lecomte (Ho = 0.14 to 0.34; [48]). These unexpected results demonstrate that even small populations may maintain adequate genetic diversity.

In our study, the high levels of genetic diversity can be attributed to the occurrence of hybridization, since genetic diversity was usually higher in sympatric than allopatric populations (Table 2, except for INF-NUM and INF-TAM). Heterozygosity values, the number of effective alleles, Shannon’s information index, and the percentage of polymorphic loci were higher in sympatric than in allopatric populations, probably as a consequence of gene flow between the two *Ocotea* species. Indeed, hybridization can leave signatures in an organism’s genome that persist over time [49,50]. Despite the high pairwise genetic differentiation occurring between the two species (Table 3), our data showed that gene flow occurs between *O. loxensis* and *O. infrafoveolata*. Allopatric populations of *O. loxensis* were composed of pure individuals, but also F1s and backcrossed hybrids towards *O. loxensis*. F1 hybrids were also detected in sympatric populations of *O. infrafoveolata*, but also in two allopatric populations, INF-NUM and INF-TAM, which were mainly composed of backcrossed hybrids towards *O. infrafoveolata* (Figure 4). Even the two allopatric populations of *O. infrafoveolata*, INF-CJS, and INF-QUH, were suggested to be composed by late hybrids. This complex evolutionary scenario could explain why *O. loxensis* can be misidentified as *O. infrafoveolata*, due to the complexity of accurate diagnostic traits even in populations that are not mixed. Hybridization and introgression can blur taxonomy and species delimitation due to the origin of intermediate morphological traits in hybrid organisms that are often not easy to distinguish from parental species [19,37,41,51].

The presence of allopatric “mosaic” populations consisting mainly of backcrosses towards *O. infrafoveolata* (e.g., INF-NUM, INF-TAM) and late-generation hybrids (e.g., INF-CJS and INF-QUH), as well as the presence of several F1 hybrids raises several hypotheses. For instance, a recent and recurrent contact between these species would explain the presence of F1s, especially because species have overlapping flowering periods [52,53,54]. *Ocotea* species are often pollinated by generalized insects such as thrips that can promote gene flow between species [55]. In addition, because these insects fly at short ranges, the occurrence of other biotic agents (and the action of wind) has been postulated to occur in *Ocotea* due to the presence of gene flow between distant populations [40]. Altogether, this would explain the existence of the current gene flow between *O. loxensis* and *O. infrafoveolata.* The prevalence of F1 hybrids has also been reported in other studies. For instance, hybrid zones of *Populus alba* and *P. tremula* are mainly composed of F1 hybrids, with genomic studies indicating selection against recombinant genotypes even under the possibility of introgression upon secondary contact [56]. The presence of F1 hybrids was also predominant and helped to increase the level of genetic differentiation and heterozygosity in mixed populations of *Ulmus rubra* and *U. pumila* [57]. Stable F1 hybrid zones were also reported in *Populus × jrtyschensis* (*P. nigra* × *P. laurifolia*) populations [58].

Although accurate data on the age of *Ocotea* trees have yet to be obtained, several individuals in these populations are at least 60 years old according to preliminary estimates based on their large stems (unpublished results). Therefore, although recent hybridizations between the two parental species might continuously produce more F1s, at least some individuals within populations would have existed long enough for post-F1 generations and backcrossed hybrids to have also been produced, supporting the genetic results found in our study. The maintenance of backcrossed hybrids and allopatric populations composed of late hybrid generations in *Ocotea* can be explained if potential adaptative traits occur in these generations, allowing them to persist through time while other less fitted hybrids disappear [49,50,59]. Our genetic data favor this hypothesis since genetic diversity was higher in sympatric than in allopatric populations. In fact, genetic diversity in allopatric populations was only lower in INF-NUM and INF-TAM populations, which were mainly composed of backcrossed hybrids towards *O. infrafoveolata.* The fitness of hybrids can be highly variable throughout generations, and the first few generations of back-crossed hybrids might indeed be less fitted than other hybrid generations, as reported in other organisms [60]. Finally, the outcomes of hybridization are often environment-dependent, with some hybrid generations being better fitted than their parents in some conditions, and less fitted in others (e.g., [61]). This is even more important in the context of environmental changes, where hybrids have the potential to adapt faster than parental populations [62].

We cannot exclude the existence of intrinsic reproductive barriers, preventing the formation of certain hybrid generations, as reported in other organisms [63,64,65]. For instance, in this study, hybrids backcrossed towards *O. infrafoveolata* do not occur in sympatric populations of *O. infrafoveolata* (Figure 4). Pre- and postzygotic barriers, which play an essential role in the formation of hybrids [66,67,68], can explain the asymmetric outcomes of hybridization in *Ocotea*. In our study, gene flow (Nm) was estimated to be more likely to occur from *O. loxensis* to *O. infrafoveolata* than in the opposite direction. In accordance, backcross generations towards *O. loxensis* were overall lower than towards *O. infrafoveolata*, suggesting incompatibility differences in the species acting as a maternal donor for the formation of hybrids. Truly, this hypothesis should be tested through artificially controlled experiments. It is also likely that backcross hybridizations towards *O. infrafoveolata* were excluded by unfavorable genetic–environmental combinations leading to unfit progeny. Habitat-mediated selection might act, excluding genotypes that are less fitted [69]. The existence of allopatric populations of *O. infrafoveolata* composed of late-generation hybrids reveals an important spatial isolation barrier between hybrids and pure parental species, contributing to the spread and persistence of these hybrids. Further, if hybridization contributes to boosting fitness in these species, it might also be linked to the high rate of species described within the tropical trees of *Ocotea*, a hypothesis that should be tested in other species.

## 4. Materials and Methods

### 4.1. Species and Population Sampling

A total of 13 populations were sampled in this study targeting 288 adult trees: 104 trees from 5 populations of *O. loxensis*, and 184 trees from 8 populations of *O. infrafoveolata*. Sampling was concentrated in the South of Ecuador, where all known populations of *O. loxensis* occur, but sampling of *O. infrafoveolata* was also performed in other areas of occurrence to better understand the patterns of gene flow (Figure 1). Sympatric vs. allopatric populations were collected using adult trees (Figure 1). In accordance with [46], individuals with a DBH higher than 5 cm were considered adult trees. Leaf samples from 20–25 adult trees were collected in each population, brought to the laboratory, and stored at −80 °C until DNA extraction.

### 4.2. DNA Extraction and nSSR Development

Total genomic DNA was extracted using the DNeasy Plant Minikit (Qiagen, Hilden, Germany) according to the manufacturer’s instructions and stored at −80 °C. Samples were first genotyped using nuclear simple sequence repeats (SSRs) previously developed for *O. rotundata* [40]. However, only 3 were polymorphic and amplified well in the species studied here (*Orot8*, *Orot21*, and *Orot22*, [40]. Due to this small number of markers, we used the extracted DNA of *O. loxensis* and *O. infrafoveolata* to develop new nuclear SSRs, using two small, inserted libraries digested with *HaeII* and *RsaI* and enriched with (CT)n sequences. Following [40], DNA fragments of each species were ligated into a p-GEM-T Easy Vector, as these were the plasmids transformed using *Escherichia coli* cells (Promega, Madison, WI, USA). In total, we obtained 60 clones in *O. loxensis* (22 from *HaeII* and 38 from *RsaI*) and 41 in *O. infrafoveolata* (20 from *HaeII* and 21 from *RsaI*), from which 48 showed a positive hybridization signal in *O. loxensis* and 36 in *O. infrafoveolata*. Positive clones were sequenced with the M13 primers under the following conditions: 3 min at 94 °C, followed by 48 cycles at 94 °C for 1 min, annealing at 53 °C for 1 min, 2 min at 72 °C, and 5 min at 72 °C. DNA sequencing was performed in both directions in a 3730 DNA Analyzer (Applied Biosystems, Foster, CA, USA). In total, 32 and 22 clones of *O. loxensis* and *O. infrafoveolata*, respectively, had readable sequences.

We used Primer 3 [70] to develop the new primers, which were first tested using 2 individuals per population of each species. SSR amplifications were performed in 15 μL reactions containing 1.25 U MyTaq DNA polymerase and 1× MyTaq Reaction Buffer (Meridian Bioscience, London, UK), 0.4 μM Primer F-FAM and R, and 100 ng of genomic DNA carried and amplified as described in [40]. Multiplexed PCR products were genotyped on an Applied Biosystems 3130XL Genetic Analyzer using 2 μL of amplified DNA, 12 μL of Hi-Di formamide, and 0.4 μL of GeneScan-600 (LIZ) size standard (Applied Biosystems, Waltham, MA, USA). Genotyping of microsatellite fragments was conducted on an AB 3500 Genetic Analyzer (Life Technologies Inc., New York, NY, USA). Allele sizes were determined using GeneMarker 3.1. (Softgenetics, State College, PA, USA). In the end, we selected 15 new nSSRs (Table 1) that amplified well in the two species, were polymorphic, and did not show any evidence of null alleles using MICRO-CHECKER v.2.2.3 [71]. These markers were used to sequence the 288 samples included in this study (Table 1). For each microsatellite locus, genetic diversity was assessed by calculating the mean number of alleles (Na), the mean number of effective alleles (Ne), the Polymorphism Information Content (PIC), Shannon’s information index (I), the mean expected heterozygosity (He), and the mean observed heterozygosity (Ho) using GenAlEx v6.51 [72]. We also tested deviation from Hardy–Weinberg Equilibrium (HWE) using the same program. In all analyses, significant values were corrected for multiple comparisons by Bonferroni correction [73].

### 4.3. Genetic Diversity and Differentiation between O. loxensis and O. infrafoveolata

The genetic diversity was assessed by calculating the total number of alleles (Ta), mean number of alleles per locus (Na), Shannon’s information index (I), mean expected heterozygosity (He), mean observed heterozygosity (Ho), inbreeding coefficient (Fis), and the percentage of polymorphic loci (PPL) using GenAlEx 6.51 [72]. We analyzed significant differences between populations and species using an ANOVA followed by a post hoc Tukey’s test (*p* < 0.05). To calculate genetic differentiation coefficient values (Fst) between populations we used ARLEQUIN (version 3.5), and also to perform the analysis of molecular variance (AMOVA) [74]. The significance of AMOVA components was analyzed by 1000 permutations.

### 4.4. Genetic Structure of Populations

To visualize the degree of the genetic structure of populations, a principal component analysis (PCoA) based on Nei’s genetic distance was constructed using GenAlEx 6.51 [72]. To understand the genetic composition of populations, STRUCTURE v.2.3.4 [75] was run from K = 1 to K = 15 to identify the best K (genetic group) value using all samples from the two species. Models were run assuming ancestral admixture and correlated allele frequencies using run lengths of 300,000 steps for each K after a burn-in of 50,000, and using 10 repetitions per K. The optimum K was determined using STRUCTURE HARVESTER [76], which identifies the optimal K based on both the posterior probability of the data for a given K and the ∆K.

### 4.5. Genetic Composition of Hybrids

The genetic composition of hybrids was tested using the Bayesian clustering method implemented in NEWHYBRIDS version 1.1.beta3 [77], which assigns individuals to 6 different classes: 2 pure parental species (*O. loxensis* and O. *infrafoveolata*), F1, F2, and late-generation hybrids, and 2 backcrosses with each parental species [77], using allopatric populations as the reference for the pure individuals (Figure 1). Because these are long-lived trees and the power of detection of late hybrid generations is limited, we treated F2s as a single group of late hybrids [78]. NEWHYBRIDS analyses were based on the same computational parameters as those conducted in STRUCTURE using a threshold of q = 0.90. Similarity coefficients between STRUCTURE and NEWHYBRIDS runs and the average matrix of ancestry membership was calculated using CLUMPP version 1.1 [79] and visualized using DISTRUCT [80]. Finally, to estimate the level of gene flow between species we used the coalescent-based program IMa2 [81], assuming a mutation rate of 10^−4^ substitutions/site/year [82]. The average generation time was set to 15 years.

## 5. Conclusions and Prospects

This study revealed the existence of past and current gene flow between *O. loxensis* and *O. infrafoveolata*, and a complex scenario ranging from the presence of F1s, backcrossed hybrids (mostly towards *O. infrafoveolata*), as well as F2s and late-generation hybrids, even in allopatric populations where only one species currently occurs. Habitat-mediated selection as well as the continuous existence of gene flow due to repeated hybridizations between these species are likely to maintain these hybrids, which seem to have adaptive potential.

Genetic diversity was usually higher in sympatric than in allopatric populations, providing a larger pool of raw genetic material for adaptive evolution in *Ocotea*. However, we should also take into consideration that long-lived species such as trees may need centuries to record both positive and negative effects. Thus, despite these high levels of genetic diversity, the very low number of populations of *Ocotea* in Ecuador, especially in the endemic *O. loxensis*, should be a sign of concern in a habitat that is undergoing increasing amounts of disturbance. In this context, management plans for the Ecuadorian forests should concentrate on in and ex situ conservation actions to maintain the genetic diversity of *Ocotea* populations and the connectivity between populations.

## Figures and Tables

**Table 1:** Characteristics of the 15 microsatellite markers used to amplify 288 samples of *Ocotea loxensis* and *O. infrafoveolata*. Na: The number of different alleles; Ne: the number of effective alleles; Ho: observed heterozygosity, He: expected heterozygosity. PIC: Polymorphism Information Content * indicates microsatellites from [40].

Locus	Primers (5′-3′)	Ta (°C)	Repeat Motif	Size Range (bp)	Accession Number	Na	Ne	Ho	He	PIC
Orot8	F: GTCGGAAACTCTACCAAAGTGA	58	(TC)8	131–140	OP428739 *	3.154	2.635	0.391	0.425	0.782
	R: CCATCCCCGTAGAGTCTCG									
Orot21	F: CGGGACTATCAGAAGGTACGT	59	(GT)22	180–185	OP428742 *	1.769	1.508	0.385	0.218	0.873
	R: TGGGTAAAAGTCTGCTGATCCT									
Orot22	F: TCCTCCTACTCCTATCTACGGA	50	(CT)13	148–155	OP428743 *	2.923	2.162	0.490	0.436	0.784
	R: ATCGTCTCTGCTATCCCTGC									
Olox1	F: TGAGGAGTAGGGAATGTCGG	58	(CTC)4	192–198	PP791920	2.538	2.022	0.884	0.492	0.905
	R: GGTACCTCCCGTAAAGTCGA									
Olox3	F: GTGGAGGTCTGCTACGCG	60	(TA)10	183–186	PP791921	2.462	1.201	0.065	0.155	0.899
	R: AAGTCCCCATAGCGATCCAG									
Olox4	F: GCTGCGAGGAGGGATGATC	60	(TTC)8	179–183	PP791922	3.000	2.085	0.326	0.405	0.803
	R: CCCGTAGTAGTAGAGTCCCG									
Olox8	F: GGTATGAGCGCCCCATCTAG	59	(TATC)6	173–177	PP791923	1.923	1.580	0.186	0.254	0.789
	R: TAAACCCGTACATCCGTCCC									
Olox9	F: CGGAGTAGAGCAATCCCCTA	59	(GC)15	179–185	PP791924	5.077	3.732	0.873	0.679	0.903
	R: CTGTATCCCCATTCCCCGAA									
Oinf2	F: GACTAGGGATCGCTGGGAC	59	(TATA)10	173–183	PP791925	2.462	2.107	0.477	0.357	0.865
	R: GTCCCCTAAATCCCGAGTCC									
Oinf4	F: GGATGTCCTGACTCGGGG	59	(AT)14	178–192	PP791926	1.385	1.385	0.385	0.192	0.788
	R: CCTCCCCAGCTCGCATATC									
Oinf5	F: TCGACGCTCCTATGGATAGC	59	(CTT)8	191–200	PP791927	4.615	3.170	0.736	0.682	0.901
	R: TCCTCACCTGTCGCACTG									
Oinf6	F: CGAGGGACCCGAGAGAGA	59	(TC)10	163–174	PP791928	2.769	1.936	0.229	0.407	0.782
	R: AGCTCTCTCTCCCTAAATCGG									
Oinf10	F: GTGACGACGCCCATATAATAGG	58	(GTTT)6	178–185	PP791929	2.769	2.117	0.492	0.463	0.885
	R: CGAAAAGGCGCGAGGTATC									
Oinf14	F: CGGAGCACTATTTTATTTAGCGT	58	(TA)16	163–174	PP791930	4.692	3.061	0.522	0.538	0.823
	R: GGGTCTACGTGTGTGTGCAT									
Oinf20	F: GGGGATTATAGGCGAGGGAG	59	(CTA)10	153–163	PP791931	3.538	1.908	0.160	0.384	0.886
	R: TCCCTCCCCGTCAATCCTAT									

**Table 2:** Genetic variation in *O. loxensis* and *O. infrafoveolata* populations. Na: The number of different alleles; Ne: the number of effective alleles; I: Shannon’s information index, Ho: observed heterozygosity; He: expected heterozygosity; Fis: inbreeding coefficient among individuals within populations; PPL: the percentage of polymorphic loci (%). Different superscript letters indicate significant differences between populations based on ANOVA followed by the post hoc Tukey’s test (*p* < 0.05). * indicates allopatric populations.

Species	Populations	N	Na	Ne	I	Ho	He	Fis	PPL
*O. loxensis*									
	LOX-SJB *	20	3.733 ± 0.384 ^a,b^	2.518 ± 0.265	0.968 ± 0.125 ^b^	0.487 ± 0.089 ^a^	0.518 ± 0.062 ^b^	0.014 ± 0.133 ^d^	93.33 ^c^
	LOX-TIR	21	4.231 ± 0.271^b^	2.696 ± 0.224	1.115 ± 0.076 ^c^	0.719 ± 0.077 ^b^	0.656 ± 0.031 ^c^	−0.042 ± 0.124 ^c^	100.00 ^a^
	LOX-CAJ	20	4.210 ± 0.279 ^b^	2.658 ± 0.206	1.112 ± 0.074 ^c^	0.700 ± 0.076 ^b^	0.692 ± 0.031 ^c^	−0.035 ± 0.123 ^c^	100.00 ^d^
	LOX-VIL	21	4.222 ± 0.271 ^b^	2.844 ± 0.224	1.149 ± 0.074 ^c^	0.741 ± 0.081 ^b^	0.619 ± 0.030 ^c^	−0.033 ± 0.134 ^c^	100.00 ^d^
	LOX-CHA	22	4.260 ± 0.279 ^b^	3.045 ± 0.231	1.196 ± 0.071 ^c^	0.730 ± 0.082 ^b^	0.643 ± 0.029 ^c^	−0.002 ± 0.132 ^c^	100.00 ^d^
*O. infrafoveolata*									
	INF-TIR	23	4.467 ± 0.376 ^b^	3.143 ± 0.268	1.232 ± 0.084 ^c^	0.749 ± 0.081 ^b^	0.647 ± 0.031 ^c^	−0.030 ± 0.135 ^c^	100.00 ^d^
	INF-CAJ	21	4.467 ± 0.376 ^b^	3.004 ± 0.280	1.177 ± 0.093 ^c^	0.733 ± 0.080 ^b^	0.623 ± 0.037 ^c^	−0.202 ± 0.129 ^b^	100.00 ^d^
	INF-VIL	23	4.333 ± 0.361 ^b^	2.994 ± 0.226	1.193 ± 0.075 ^c^	0.781 ± 0.089 ^b^	0.641 ± 0.025 ^c^	−0.197 ± 0.153 ^b^	100.00 ^d^
	INF-CHA	21	4.333 ± 0.374 ^b^	2.977 ± 0.228	1.188 ± 0.076 ^c^	0.749 ± 0.081 ^b^	0.639 ± 0.025 ^c^	−0.199 ± 0.143 ^b^	100.00 ^d^
	INF-NUM *	22	4.267 ± 0.396 ^b^	3.097 ± 0.250	1.206 ± 0.084 ^c^	0.730 ± 0.081 ^b^	0.650 ± 0.025 ^c^	−0.170 ± 0.153 ^b^	100.00 ^d^
	INF-TAM *	24	4.200 ± 0.355 ^b^	3.068 ± 0.242	1.188 ± 0.076 ^c^	0.794 ± 0.070 ^b^	0.650 ± 0.023 ^c^	−0.268 ± 0.136 ^b^	100.00 ^d^
	INF-CJS *	25	2.467 ± 0.413 ^a^	1.894 ± 0.164	0.626 ± 0.115 ^b^	0.501 ± 0.114 ^a^	0.296 ± 0.065 ^a^	−0.798 ± 0.069 ^a^	73.33 ^b^
	INF-QUH *	25	2.067 ± 0.396 ^a^	1.653 ± 0.177	0.454 ± 0.123 ^a^	0.525 ± 0.131 ^a^	0.288 ± 0.073 ^a^	−0.848 ± 0.063 ^a^	53.33 ^a^

**Table 3:** Genetic differentiation coefficient Fst (below diagonal) between *O. loxensis* and *O. infrafoveolata* populations. Color degree indicates the level of genetic differentiation. Population codes follow Figure 1. * indicates allopatric populations.

LOX-SJB *	LOX-TIR	LOX-CAJ	LOX-VIL	LOX-CHA	INF-TIR	INF-CAJ	INF-VIL	INF-CHA	INF-NUM *	INF-TAM *	INF-CJS *	INF-QUH *	
0													LOX-SJB *
0.110	0												LOX-TIR
0.108	0.038	0											LOX-CAJ
0.104	0.028	0.043	0										LOX-VIL
0.113	0.046	0.047	0.030	0									LOX-CHA
0.320	0.321	0.334	0.315	0.290	0								INF-TIR
0.342	0.341	0.355	0.334	0.311	0.012	0							INF-CAJ
0.333	0.337	0.351	0.331	0.306	0.009	0.009	0						INF-VIL
0.363	0.36	0.375	0.353	0.329	0.017	0.013	0.010	0					INF-CHA
0.344	0.337	0.352	0.331	0.310	0.013	0.015	0.011	0.008	0				INF-NUM *
0.357	0.354	0.367	0.349	0.327	0.025	0.024	0.021	0.011	0.017	0			INF-TAM *
0.502	0.514	0.531	0.502	0.482	0.204	0.200	0.204	0.199	0.196	0.211	0		INF-CJS *
0.520	0.534	0.550	0.521	0.501	0.273	0.270	0.277	0.276	0.269	0.289	0.302	0	INF-QUH *

**Table 4:** Analysis of molecular variance (AMOVA) for *O. loxensis* and *O. infrafoveolata*, considering all samples, only *O. loxensis* populations and only *O. infrafoveolata* populations.

Populations	df	SS	% Variance
All samples			
Among populations	12	1147.090	39%
Within populations	275	956.500	61%
*O. loxensis*			
Among populations	4	3.660	6%
Within populations	104	46.500	94%
*O. infrafoveolata*			
Among populations	7	804.600	41%
Within populations	176	1180.796	59%

## Data Availability

Data will be made available on request.
